# The Short Version of the Italian Maastricht Vital Exhaustion Questionnaire (MVEQ): Psychometric Properties and Relationships with Anxiety, Depression, and Stress in a Community Sample of Older Adults

**DOI:** 10.1007/s10880-024-10007-7

**Published:** 2024-02-23

**Authors:** Marta Spinoni, Andrea Zagaria, Cristiano Violani, Caterina Grano

**Affiliations:** Department of Psychology, Sapienza University of Rome, Via Dei Marsi 78, Rome, 00185 Italy

**Keywords:** Psychometric properties, Maastricht vital exhaustion questionnaire, Vital exhaustion, Validation study

## Abstract

Vital Exhaustion (VE) refers to a physical and mental state of excessive fatigue, feelings of demoralization, hopelessness, and increased irritability. The short form of the Maastricht Vital Exhaustion Questionnaire (MVEQ) is a widely used measure to assess VE. Despite its utility is broadly recognized, the validity and reliability of the scale have yet to be examined in the Italian context. The present study aimed to evaluate the psychometric properties of the shortened MVEQ in a community sample of Italian older adults. A total of 722 older adults (M_age_ = 72.97, SD = 7.71; 60.4% females) completed the MVEQ, as well as other self-report questionnaires assessing anxiety, depression and stress in order to evaluate the criterion-related validity of the scale. A confirmatory factor analysis (CFA) was conducted to examine the original MVEQ latent structure. Internal consistency was assessed through model-based omega coefficient. Test-retest reliability was examined by re-administering the MVEQ after three months to a subsample of 568 participants. Factorial invariance tests across gender were conducted by means of multi-group CFAs. The one-factor model showed an acceptable fit to the data. The MVEQ yielded a reliable total score (ω = 0.822) and showed moderate-to-large correlations with measures of anxiety, depression, and stress (r range 0.30 to 0.75, *ps* < 0.001). Test-retest reliability was supported by an Intraclass Correlation Coefficient (ICC) of 0.661. Lastly, the scale was factorially invariant across gender. Overall, the MVEQ provided evidence of reliability and criterion-related validity in a sample of Italian older adults and may be useful for both clinical and research practices.

## Introduction

Vital Exhaustion (VE) refers to a state of excessive psychophysical fatigue, feelings of demoralization, hopelessness, and increased irritability (Appels et al., 1987; Appels, 1990). It is often considered a form of adaptation to prolonged distress (Bennet et al., 2008) and has been widely studied in relation to adverse health outcomes, proving to be an independent risk factor for the development and progression of cardiovascular diseases (Cohen et al., 2017; Balog & Thege, 2018). Indeed, VE has been related to diminished cortisol concentration (Strahler & Fischer, 2018; Nicolson & van Diest, 2000) increased risk of type 2 diabetes (Strikwerda et al., 2021), augmented lipid metabolism (Igna et al., 2011), reduced fibrinolytic capacity (Von Känel et al., 2009; Kop et al., 1998), decreased parasympathetic activity (Watanabe et al., 2002), and higher levels of inflammatory cytokines (Hoekstra et al., 2013), all mechanisms involved in cardiovascular diseases (Kubzansky et al., 2005). Moreover, two recent meta-analyses showed an augmented risk for cardiac events between 1.50 and 2.03 in people with increased levels of VE (Cohen et al., 2017; Frestad & Prescott, 2016). The established positive association between vital exhaustion and coronary heart diseases led some authors to suggest that VE should be included in the European Society of Cardiology (ESC) guidelines (Visseren et al., 2021) and in the Systematic Coronary Risk Evaluation (SCORE) algorithm (Graversen et al., 2016).

The most common measure used for assessing VE is the Maastricht Vital Exhaustion Questionnaire (MVEQ), a 21-item scale, originally developed by Appels and colleagues (1987) to prospectively identify cases of cardiac events both in healthy subjects and in coronary heart disease patients. Items were derived from coronary patients’ self-descriptions of the period preceding myocardial infarction (e.g., “My body was like a battery that is losing its power”; “I had no more power. That made me feel miserable”), and the instrument was originally validated on a sample of 3877 male adults aged 39 to 65 (Appels et al., 1987). In the original validation study, it has been suggested to consider MVEQ as a unidimensional measure, with one single composite score (0 to 42) reflecting the overall level of vital exhaustion (Appels et al., 1987). In the above mentioned study, a high internal consistency was reported (α = 0.89) and with respect to criterion-related validity, elevated scores of VE were predictive of myocardial infarction with a relative risk of 2.3 to 4.2 years of follow-up. This version of MVEQ was extensively utilized in the scientific literature and has been adapted into multiple languages, including German (Kop et al., 1996), Greek (Anagnostopoulou & Kioseoglu, 2002), Spanish (Bagés et al., 2000), Finnish (Keltinkangas-Jirvinen et al., 1996), and Swedish (Kristenson et al., 1998). The scale has been employed in numerous longitudinal studies, evidencing that MVEQ scores are independent risk factors for incident and recurrent cardiac events (Williams et al., 2010), strokes (Schuitemaker et al., 2004), coronary heart diseases (Frestad & Prescott, 2016), and all-cause mortality (Prescott et al., 2003).

However, some studies have reported limited replicability of the unidimensional factor structure of the original version of the scale, with different factorial solutions ranging from one to multiple dimensions. For example, two principal component analyses (PCA) by Smith and colleagues (2009) and by Pedersen and colleagues (2007) reported respectively four (fatigue, cognitive/affective depressive symptoms, sleep difficulties, and lack of concentration) and two (depression and fatigue) components; while McGowan and colleagues (2004), using a PCA followed by an orthogonal rotation, found four dimensions, namely fatigue, depression, lack of concentration and sleeping problems. Moreover, some authors criticized the potential overlap between VE and depression (Luepker & Schulz, 2015; Bianchi et al., 2017), failing to reveal depression and VE as two distinct factors (Wojciechowski et al., 2000).

In trying to solve the factorial instability and the lack of discriminant validity between VE and depression, a shortened 9-item version deriving from the 21-item MQ and containing only the most representative items for the construct of vital exhaustion was developed (Kopp et al., 1998, 2010). In a study conducted on the general Hungarian population with a sample of 12,640 over the age of 16, the authors reported strong correlations with the full-length version (*r* = .94) and a good internal consistency coefficient (α = 0.83) (Kopp et al., 1998). Its factor structure showed to be more replicable demonstrating a one-factor model across studies consistently (e.g., Schnorpfeil et al., 2002). The shortened version was used in several studies reporting strong associations with both physical and mental health outcomes in different settings, comparable to the ones of the original scale (von Känel et al., 2004; Kopp et al., 2010; Ramsey et al., 2014; Prescott et al., 2003; Balog & Thege, 2018). For example, the MVEQ short version scores were associated with increased D-dimer levels in stress-induced conditions (von Känel et al., 2009), with a higher risk of coronary artery disease progression (Deter et al., 2021) and type 2 Diabetes (Strikwerda et al., 2021). Moreover, this instrument was used to evaluate the association between VE and an increased risk for Alcohol Use Disorder (Just-Østergaard et al., 2018), worst quality of life (Kotova et al., 2021) and reduced levels of well-being in general (Rafael et al., 2014).

The present investigation aimed to adapt the short 9-item version of the Maastricht Vital Exhaustion Questionnaire into Italian and test its psychometric properties in a community sample of Italian older adults, by analyzing its factorial structure, composite and test-retest reliability, criterion-related validity and measurement invariance across gender.

## Methods

### Participants and Procedures

We recruited a community sample of 772 Italian older adults, aged 60–96, residing in the central and southern regions of Italy. Participants were directly and opportunistically recruited in public locations, took part on a voluntary basis, and were not remunerated. After explaining the procedure of the study and signing the informed consent form, respondents were asked to complete a group of self-report questionnaires. Following the initial assessment, the same participants were contacted after a period of three months to complete the MVEQ for a second time in order to assess the test-retest reliability of the scale. All participants gave fully informed written consent to participate in the study. Anonymity was guaranteed by using alphanumeric codes.

### Measures

#### Short Version of the Maastricht Vital Exhaustion Questionnaire (MVEQ)

The shortened Maastricht Vital Exhaustion Questionnaire (MVEQ) was employed to assess vital exhaustion (Appels et al., 1987; Kopp et al., 1998). The nine items are rated with 0 (“*no*”), 1 (“*don’t know*”), or 2 (“*yes*”), resulting in a total score ranging from 0 to 18, where higher scores indicate greater levels of vital exhaustion. Participants were asked to report whether they generally experienced each of the feelings indicated in the items. Examples of items include “*Do you often feel tired?*” and “*Do you feel weak all over?*”. For the purpose of this study, following international guidelines for psychological test adaptation (Hambleton, 1994), the MVEQ was translated from English into Italian by an expert fluent in both languages. Subsequently, an independent bilingual back-translated the Italian version into English to verify conceptual equivalence. The two versions were compared by an independent researcher expert in the field, and no further adjustments were required. The content of the items is presented in Table 1. The Italian version of the questionnaire employed in this study can be requested by contacting the corresponding author.

**Table 1:** Item-level descriptive statistics of the shortened version of the Maastricht Vital Exhaustion Questionnaire

	Response Frequencies (%)		
Items	0	1	2
1. Do you often feel tired?	33.3	26.9	39.8
2. Do you often have difficulty falling asleep?	41.5	29.6	28.9
3. Do you wake up repeatedly during the night?	41.4	21.1	37.6
4. Do you feel weak all over?	42.2	34.1	23.7
5. Do you lately feel more listless than before?	38.5	25.1	36.3
6. Do little things irritate you more lately than you used to?	41.2	30.2	28.6
7. Do you sometimes feel that your body is like a battery that is losing its power?	40.7	24.1	35.2
8. Do you feel dejected?	37.8	34.3	27.9
9. Do you ever wake up with a feeling of exhaustion and fatigue?	43.1	25.7	31.2

#### Geratric Anxiety Inventory (GAI)

To assess the criterion-related validity of the MVEQ, the Geriatric Anxiety Inventory (GAI, Pachana et al., 2007; Italian version Ferrari et al., 2017) was employed. The GAI consists of 20 Agree/Disagree items for the assessment of anxiety symptomatology in the older adult population, with higher scores reflecting higher levels of anxiety symptoms. Examples of items are: “I worry a lot of the time”; “I find it hard to relax”. Cronbach’s alpha in the present investigation was 0.931.

#### Geriatric Depression Scale (GDS)

To assess the criterion-related validity of the MVEQ, the Geriatric Depression Scale (GDS, Yesavage et al., 1983; Italian version Galeoto et al., 2018) was employed. The GDS is a self-report measure comprising 30 items assessing depressive symptomatology in older adults. Participants were asked to indicate the presence of depressive symptoms by responding with either ‘Yes’ or ‘No’ with higher scores reflecting higher levels of depressive symptomatology. Examples of items are: *“Do you feel that your life is empty?”; “Do you prefer to stay at home, rather than going out and doing new things?”.* Cronbach’s alpha in the present study was 0.975.

#### Perceived Stress Scale (PSS)

To assess the criterion-related validity of the MVEQ, the Perceived Stress Scale (PSS, Cohen et al., 1983; Italian version Mondo et al., 2021) was administered. PSS is a 14-item self-report instrument evaluating individuals’ appraisal of their life as stressful over the past month. Examples of items are: “*Felt nervous or stressed*”; “*Felt able to control irritations in your life*”. Participants respond to each item on a five-point Likert scale ranging from 0 (“Not at all”) to 4 (“Very often”), rating how often they had experienced these feelings, with higher scores reflecting higher levels of perceived stress. Cronbach’s alpha in the present sample was 0.787.

### Data Analysis

Data were analysed using Jamovi (https://www.jamovi.org) and Mplus v. 8.6 (Muthén & Muthén, 1998–2017).

In order to test the original one-factor structure of the scale, a confirmatory factor analysis (CFA) was conducted. As the response format for MVEQ items includes only three ordered options, data were treated as ordinal and the factor model was fitted to the inter-item polychoric correlations using WLSMV (see Muthén & Muthén, 1998–2017), a robust weighted least squares estimator recommended to deal with ordinal observed indicators of underlying continuous latent variables (Flora & Curran, 2004). Polychoric-based factor analysis can be conceived as a different parameterization of the multidimensional normal-ogive graded response model (Ferrando & Lorenzo-Seva, 2013). Following a multifaceted approach (Tanaka, 1993), several goodness-of-fit indices were computed to evaluate how well the model fitted the observed data (Wang & Wang, 2020; Browne & Cudeck, 1993): root mean square error of approximation (RMSEA; ≤ 0.08 indicates moderate fit); comparative fit index and Tucker-Lewis index (CFI and TLI, respectively; ≥ 0.90 indicates acceptable fit); and standardized root mean squared residual (SRMR; ≤ 0.08 indicates good fit). Due to the well-established sensitivity of the chi-square test to sample sizes (e.g., Kline, 2011), it was reported but not employed as a measure of model fit.

Model-based omega coefficients were computed to examine the composite reliability of the scale (e.g., Flora, 2020). Differently from Cronbach’s alpha, omega is derived from the model-based factor loadings and does not assume equal factor loadings (a condition known as essentially tau-equivalence; Flora, 2020). To produce unbiased estimates of reliability for ordinal data, Green and Yang’s (2009) formula for categorical omega (ω_cat_) was employed. Moreover, in order to account for potential error covariances, the denominator for the omega calculation included the model-implied variance of the total score (Flora, 2020). Omega ranges from 0 to 1, with values higher than 0.70 indicating acceptable internal consistency in non-exploratory research (Hair et al., 2014). Afterwards, test-retest reliability was examined by re-administering the scale after 3 months to a subset of 568 participants and by calculating an intraclass correlation coefficient (ICC) through a two-way mixed-effects model based on single measures and absolute agreement (Koo & Li, 2016). ICC was interpreted following Koo & Li’s (2016) benchmarks: poor (< 0.5), moderate (between 0.50 and 0.75), good (between 0.75 and 0.90), and excellent (> 0.9).

As a further step, criterion-related validity was examined by computing zero-order correlations between the MVEQ and the GDI, GAI, and PSS. We adopted Cohen’s (1988) benchmarks of *r* = .10, 0.30, and 0.50 to interpret observed effect sizes as small, medium, or large, respectively.

Eventually, multi-group confirmatory factor analyses (MG-CFA) were conducted with the aim of testing the factorial invariance of the scale across gender. Following Meredith’s (1993) framework, we compared a series of nested models, each with increasingly stringent constraints. Namely, three levels of factorial invariance were examined: configural invariance (i.e., whether the same factorial structure held across gender), metric invariance (i.e., equality of factor loadings across gender), and scalar invariance (i.e., equality of factor loadings and thresholds across gender). To evaluate the tenability of the imposed constraints, these nested models were compared by calculating differences in CFI, TLI, and RMSEA. Specifically, changes in CFI and TLI of less than 0.01, accompanied by a change in RMSEA lower than 0.015, suggest that the more constrained and parsimonious model ought to be preferred (Cheung & Rensvold, 2002; Chen, 2007).

## Results

### Descriptive Statistics of the Sample

The sample consisted of 722 older adults, aged 60–96 (M_age_ = 72.97, SD = 7.71). Among them, 60.4% were females and 39.6% were males. Most of the sample (40.5%) had a primary-school diploma, 27.3% had a high-school diploma, 20.3% had a middle-school diploma, 7.2% had a bachelor’s or master’s degree, and 4.9% did not report any information about their education status. Regarding marital status, 67.1% of the sample were married, while 32.6% were not engaged in a relationship (i.e., single, divorced, widowed or never married). Table 2 summarizes the socio-demographic characteristics of the sample. The mean score of the MVEQ reported by the sample was 8.24 (SD = 4.82), ranging from 0 to 18. The 25th percentile fell at 4, while the 75th percentile was found at 12.

**Table 2:** Socio-demographic characteristics of participants (n = 722)

Variable	Mean (SD) or N (%)
*Age*	72.97 (7.71)
*Gender* Female Male	436 (60.4%) 286 (39.6%)
*Education* Primary School High School Middle school Bachelor’s or master’s degree Missing	292 (40.5%) 197 (27.3%) 146 (20.3%) 51 (7.2%) 36 (4.9%)
*Marital Status* Married Non-engaged	485 (67.1%) 236 (32.6%)

### Factorial Structure

**Fig. 1:**
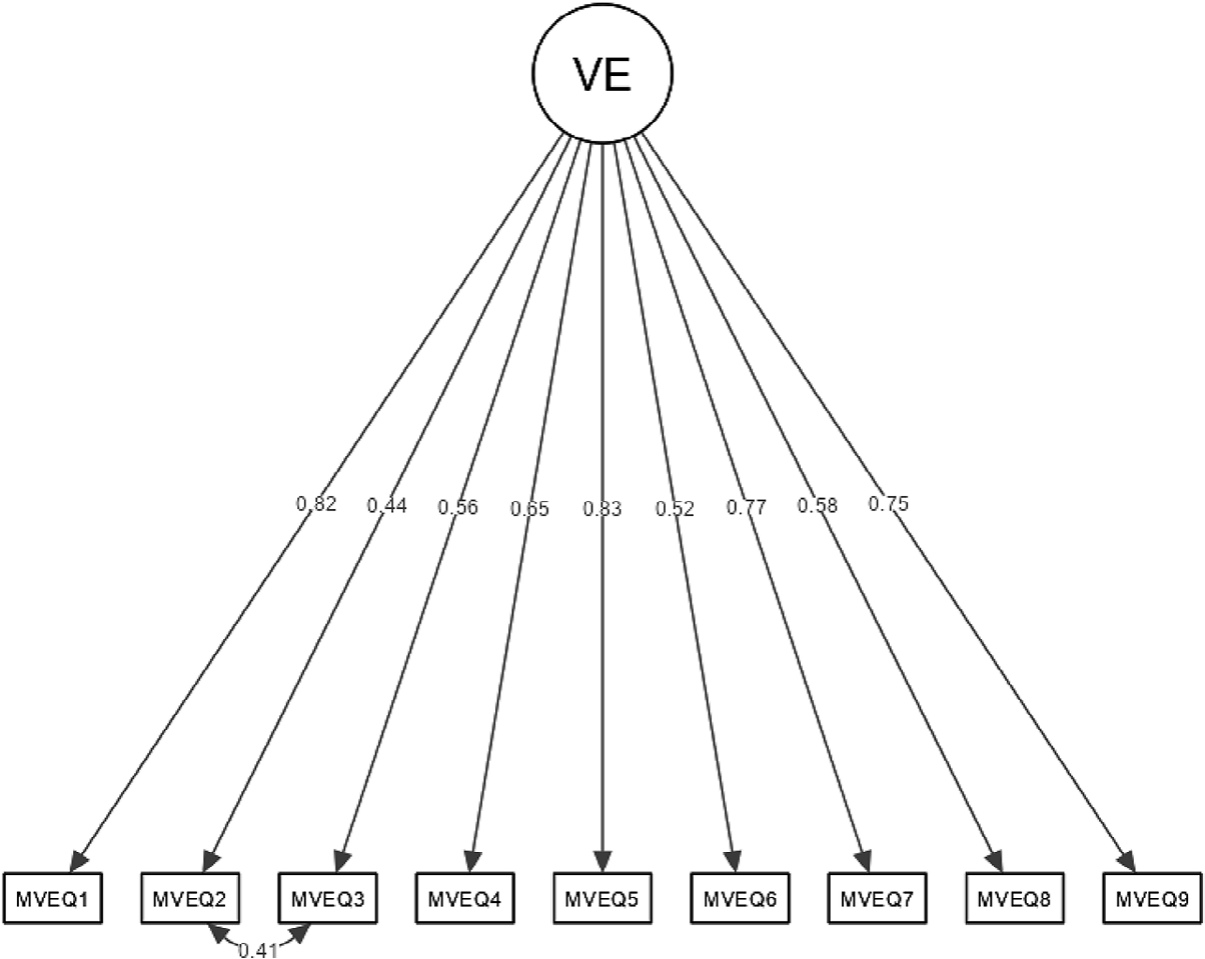
Confirmatory factor analysis of the MVEQ. *Notes* Values are reported in a completely standardized metric. All factor loadings were statistically significant at *p* < .001. Abbreviations, MVEQ, Maastricht Vital Exhaustion Questionnaire, VE, vital exhaustion

A confirmatory factor analysis (CFA) was conducted to test the hypothesised one-factor structure of the scale. Item #2 (“Do you often have trouble falling asleep?”) and Item #3 (“Do you wake up repeatedly during the night?”) of the MVEQ are thought to measure the same facet of VE, i.e., sleep difficulties, and had an adjacent position in the scale (e.g., Weijters et al., 2009). To take into account this potential extra source of item covariance (Marsh, 1996), we a-priori allowed their residuals to covary. The CFA model exhibited an acceptable fit to the empirical data: *x*^2^ (26) = 147.470, *p* < .001; CFI = 0.970; TLI = 0.959; RMSEA = 0.080; SRMR = 0.041. Standardised factor loadings were all higher than 0.40 and therefore interpreted as salient (e.g., Kline, 2011), ranging from 0.438 to 0.833 (*ps* < 0.001). The factorial structure is graphically represented in Fig. 1.

### Reliability

The coefficient omega (ω_cat_) was 0.820, highlighting good internal consistency of the scale (Hair et al., 2014). In other words, 82% of the total-score variance was due to the single factor, thus suggesting that a total score of these nine items reliably measures a VE common factor. Moreover, the test-retest reliability of the MVEQ was evaluated through the intraclass correlation coefficient (ICC) using a two-way mixed-effects model. Specifically, we found an ICC of 0.661, indicating moderate stability over three months (Koo & Li, 2016).

### Criterion-Related Validity

Criterion-related validity was supported by statistically significant correlations ranging from nearly moderate to large in size between the MVEQ and measures of depressive symptoms (GDS; *r* = .749, *p* < .001), anxiety symptoms (GAI; *r* = .322, *p* < .001), and perceived stress (PSS; *r* = .295, *p* < .001).

### Measurement Invariance

Factorial invariance tests across gender were conducted by means of multi-group confirmatory factor analysis (MG-CFA) using the WLSMV estimator. A first examination of the configural model resulted in an unsatisfactory fit to the data. More specifically, an exploration of the modification indices suggested freely estimating error covariance between Item#5 (“Do you lately feel more listless than before?”) and Item#1 (“Do you often feel tired?”) solely for the male group. Although much variance in these indicators was explained by the latent dimension of VE (as highlighted by the standardized loadings higher than 0.6), this covariance may be justified by the similarity in content of the items, further enhanced by the Italian translation of the scale, which refers to a common theme of sluggishness and fatigue. As pointed out by Wang and Wang (2020, p.258), *“the baseline model of different groups that will be integrated in the configural model must be similar, but it is not necessary for them to be completely identical”* (see also Bentler, 2006; Byrne et al., 1989). In fact, the pattern of error covariances may vary across groups without impacting the number of factors or the pattern of factor loadings (Wang & Wang, 2020; Yu et al., 2021). When the model was respecified to include this error covariance, the same factorial structure held across groups, thereby supporting configural invariance: *x*^2^ (51) = 158.457, *p* < .001; CFI = 0.974; TLI = 0.963; RMSEA = 0.076; SRMR = 0.045. By imposing constraints on factor loadings, we observed improvements in both CFI, TLI, and RMSEA, providing support for metric invariance. Lastly, introducing constraints on items’ thresholds did not result in a significant decline of model fit, suggesting the plausibility of the scalar invariance model: ΔCFI = 0.003, ΔTLI = 0, ΔRMSEA = 0.001. Invariance results are summarised in Table 3. After establishing scalar invariance, a comparison between latent means was conducted defining males as the reference group (see e.g., Wang & Wang, 2020). Findings showed that males and females reported similar levels of VE (Standardized Mean Difference = 0.095, *p* > .05).

**Table 3:** Factorial invariance tests across gender

Model	χ2 (DF)	RMSEA	CFI	TLI	SRMR	Comparison	ΔRMSEA	ΔCFI	ΔTLI
1.Configural invariance	158.457 (51)	0.076	0.974	0.963	0.045				
2.Metric invariance	157.185 (59)	0.068	0.976	0.971	0.046	2 vs. 1	-0.008	0.002	0.008
3.Scalar invariance	175.944 (67)	0.067	0.973	0.971	0.048	3 vs. 2	-0.001	-0.003	0

*Note* WLSMV was employed as the parameter estimation method

## Discussion

The main objective of this study was to examine the psychometric properties of the shortened version of the Maastricht Vital Exhaustion Questionnaire (MVEQ) in assessing vital exhaustion among a community sample of older Italian adults. The study focused on analyzing the factorial structure, composite and test-retest reliability, criterion-related validity, and measurement invariance across gender.

Confirmatory factor analysis (CFA) confirmed the unidimensional structure of the MVEQ, which adequately reproduces the inter-item correlations and exhibited a satisfactory fit to the empirical data. All standardized factor loadings, which serve as indicators of this latent dimension, were substantial in magnitude, exceeding 0.40 and thereby attesting to their salience (e.g., Kline, 2011).

The scale exhibited a good internal consistency, as indicated by an omega coefficient greater than 0.80, which suggests that a large proportion of the total-score variance was due to the single underlying construct. This finding aligns with the internal consistency highlighted in the study conducted by Kopp and colleagues (Kopp et al., 1998). Furthermore, the instrument showed adequate stability over time. Specifically, after re-administering the MVQE to a subsample of 568 individuals, the two-way mixed-effects ICC of 0.66 highlighted moderate test-retest reliability over three months (Koo & Li, 2016).

Furthermore, gender factorial invariance was established across all levels, including configural, metric, and scalar. Configural invariance implies that the same pattern of free and fixed loadings is tenable for both males and females. Metric invariance suggests that the strength of the relationships between the VE common factor and the observed indicators are equivalent for males and females (i.e., equal true score variances). Finally, scalar invariance implies that the thresholds for each item are invariant across genders. These findings ensure that the factorial structure of the MVEQ can be generalized across males and females, providing solid support for future research to conduct proper gender comparisons in the scores of VE (e.g., latent means comparisons).

As a further step, criterion-related validity was evaluated by correlating the MVEQ scores with several external variables (i.e., criteria) considered to be theoretically related to VE. Findings showed that VE scores significantly correlated with anxiety symptoms, depressive symptomatology, and perceived stress. The correlations were moderate to large in size (Cohen, 1988).

Concerning depressive symptoms, a very high correlation between MVEQ and GDS emerged, highlighting that the two constructs shared 56% of the variance. This suggests that there is some overlap between the two constructs, even though nearly half of the variance is not shared, which also implies that there are notable differences. In the literature, the possible overlap between depression and VE had already emerged (McGowan et al., 2004; Luepker & Schulz, 2015; Bianchi et al., 2017; Wojciechowski et al., 2000). In particular, it was hypothesized that vital exhaustion could be conceptualized as the same construct of the somatic/affective dimension of depression (Vroge et al., 2012). However, several studies have demonstrated that, despite the partial conceptual similarity with other psychosocial constructs, VE is a distinct phenomenon, confirming its unique impact as an independent factor for cardiovascular diseases (Kopp et al., 1998; Kudielka et al., 2004; Balog et al., 2017). For example, in a study by Balog and Thege (2018), VE remained a significative predicting variable for the reoccurrence of vascular events even after controlling for depression, anxiety, and hostility. In another study, feelings of fatigue were the strongest predictors for myocardial infarction, even when controlled for symptoms indicative of depressive affect, while, by contrast, depressive symptoms and irritability lost their predictive power when adjusted for fatigue (Appels et al., 2000).

Moreover, the meaningful association with perceived stress is consistent with the scientific literature (Kudielka et al., 2004; Schoch et al., 2018). Indeed, several studies consider vital exhaustion as a potential consequence of long-term chronic stress (Koertge et al., 2002; Noser et al., 2018). Previous studies highlighted the dysregulation of the hypothalamic-pituitary-adrenocortical (HPA) axis to be one of the pathogenic mechanisms in fatigue symptoms (Demitrack et al., 1991; Scott & Dinan, 1998), showing significant associations between VE and physiological signals of altered chronic stress system e.g., alterations in cortisol levels (Strahler & Fischer, 2018; Nicolson & van Diest, 2000), dysfunction in catecholamine response (van Doornen & van Blokland, 1989; Scott et al., 2007), and sympathovagal imbalance (Strahler & Fischer, 2018; Watanabe et al., 2002; Keltinkangas-Jirvinen et al., 1996). The nearly moderate correlation with the PSS can be explained by the fact that although both measures somehow assess the experience of stress, one assesses the degree to which individuals perceive stressful situations in their lives (Cohen et al., 1983), and the other evaluates the signs and symptoms expressions of excessive stress and fatigue (Kopp et al., 2010).

Lastly, the correlation with anxious symptomatology is consistent with previous studies evidencing that VE and anxiety are often associated with predicting worse psychophysical outcomes (Rafael et al., 2014; Qiu et al., 2012). Indeed, in a study on coronary artery disease patients, anxiety symptoms and VE were found to be significant predictors of subjective quality of life measures (Rafael et al., 2014). Moreover, the comorbidity between anxiety, depression symptomatology, and VE was significantly associated with pro-coagulant markers in depressed patients with cardiovascular diseases (Deter et al., 2021).

### Limitations

Although the present study provides valuable insights into the psychometric properties of the MVEQ, several limitations should be acknowledged. First, the generalizability of the findings may be limited as the sample only included older adults. Future research is needed to evaluate the psychometric properties of the scale in the general population. Secondly, the present study was conducted on a non-clinical sample, limiting the generalizability of the findings to clinical populations. Further research is needed to examine the psychometric properties of the MVEQ in clinical populations in Italy (e.g., patients with a diagnosis of major depressive disorder). Another limitation pertains to the response choices of the MVEQ. The original authors (Appels et al., 1987) developed the scale by rating each item on a three-point ordinal scale, namely assigning a score of 0 to the “No” option, a score of 1 to the “I don’t know” option, and a score of 2 to the “Yes” option. During the scale administration, participants were explicitly instructed to approach the three response options in an ordinal manner, with particular emphasis on selecting the middle response option when symptoms were experienced without high frequency and intensity. Importantly, no participant involved in the present study raised concerns about understanding the instructions. However, future investigations could explore an alternative scoring method, such as a dichotomous (Yes/No) response format, thus examining any discrepancies between the two approaches. Finally, this study did not assess the discriminant validity of the scale, highlighting the need for future research to evaluate the MVEQ’s ability to distinguish between vital exhaustion and other theoretically unrelated constructs.

## Conclusion

Despite these limitations, our study has several strengths. Firstly, we enrolled a large sample, enhancing the external validity and generalizability of the findings. Secondly, we employed a range of distinct criterion measures, each chosen to evaluate a different aspect of the construct targeted by the MVEQ, thereby strengthening the robustness of our findings regarding criterion-related validity. Thirdly, we included a test-retest design, enabling an assessment of the stability of the scale over time. Finally, it is worth emphasizing that the abbreviated version of the questionnaire is also advantageous, considering that short questionnaires offer obvious benefits in terms of reduced burden and cost, resulting in less participant fatigue (Cerolini et al., 2022). In conclusion, the psychometric properties of the Italian version of the shortened MVEQ were found to be satisfactory. These results suggest that the test scores can be considered valid and reliable for evaluating vital exhaustion in a sample of Italian older adults.

## Data Availability

The data are available from the corresponding author upon reasonable.
